# Conversion therapy for unresectable intrahepatic cholangiocarcinoma using gemcitabine plus S-1 combined with PD-1 inhibitors: a case report

**DOI:** 10.3389/fonc.2024.1476593

**Published:** 2025-01-15

**Authors:** Shuangying Zhao, Xiaodong Zhang, Jialiang Luo, Huanjun Yan, Jianlei Zhang, Rongfeng Lin, Kelei Zhu

**Affiliations:** ^1^ Department of Hepatopancreatobiliary Surgery, The Affiliated People’s Hospital of Ningbo University, Ningbo, China; ^2^ Health Science Center, Ningbo University, Ningbo, China

**Keywords:** intrahepatic cholangiocarcinoma, conversion therapy, case report, immunotherapy combined therapy, PD-1

## Abstract

Intrahepatic cholangiocarcinoma (iCCA) is a highly malignant tumor of the liver and gallbladder, which is usually diagnosed at an advanced stage and the opportunity for surgery is lost. Therefore, conversion therapy is important to convert the iCCA into a resectable state. In recent years, the conversion protocol of immuno-chemotherapy has been applied for advanced liver cancer. However, little has been reported about iCCA conversion therapy. The aim of this report is to present the results of conversion therapy with Gemcitabine plus S-1 (GS) combined with PD-1 inhibitors (Zimberelimab) in a 74-year-old female IIIB iCCA patient. After 6 cycles of conversion therapy, enhanced CT showed that the patient’s tumor had shrunk to nearly half its original size, making radical resection possible. Postoperative pathology showed a complete pathological response. This provides a new way to convert advanced iCCA into resectable state.

## 1 Introduction

Intrahepatic cholangiocarcinoma (iCCA) is a rare liver neoplaisa with high-grade malignancy. Studies have shown that in recent years, the incidence and mortality of this condition has been increasing worldwide year by year (1), with a five-year survival of around 15%~40% (2). Surgical resection is preferred in the current treatment options, however, more than two-thirds of the patients experience recurrence within a short-term after surgical resection, which are related to positive surgical margins, lymph node metastasis, vascular invasion and multiple lesions (3). Nevertheless, the majority of patients with iCCA are already in the advanced stage of disease at the time of confirmed diagnosis, when radical resection surgery are difficult to perform. For those patients, it is possibility that unresectable advanced iCCA will transition into the resectability status, by being treated with systemic or locoregional therapies.

Currently, gemcitabine/cisplatin combination therapy is considered the first-line treatment for advanced iCCA (4). Meanwhile, studies had found that iCCA is characterized by abundant stroma, including cancer associated fibroblasts, endothelial, and immune cells which contribute to anti-cancer immunity (5). Therefore, immunotherapy could be useful for such malignancy. In addition, growing evidence has also confirmed the benefits of immunotherapy combined with chemotherapy or targeted therapy in advanced liver cancer (6, 7).

**Figure 1:**
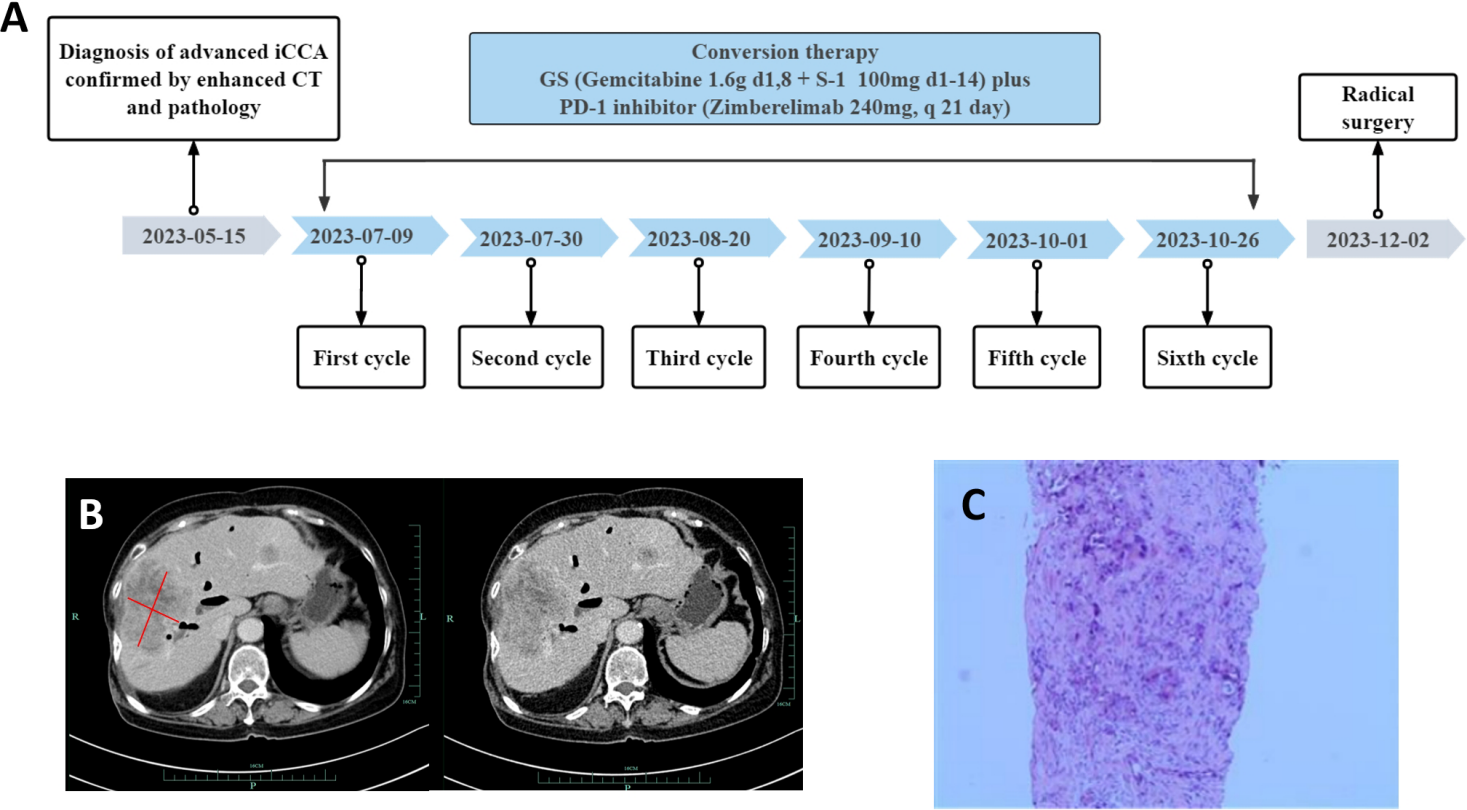
Timeline, enhanced computed tomography (CT) scan at the time of diagnosis and liver puncture pathological findings. **(A)** Showing the course of initial diagnosis, medication, and surgery. **(B)** Showing a large hypodense mass about 8.3*6.4cm size in the right lobe of the liver. **(C)** Liver puncture pathology was suggestive of poorly differentiated adenocarcinoma with necrosis.

This report presents the results of a new combined regimen with Gemcitabine plus S-1 (GS) combined with PD-1 inhibitors (Zimberelimab) for advanced iCCA, that successfully undergo curative resection, after a conversion for resectable status. The postoperative specimen showed pathological complete response according to the Response Evaluation Criteria in Solid Tumors (version 1.1). The episode of care for this patient is summarized in **Figure 1A**.

## 2 Case description

**Figure 2:**
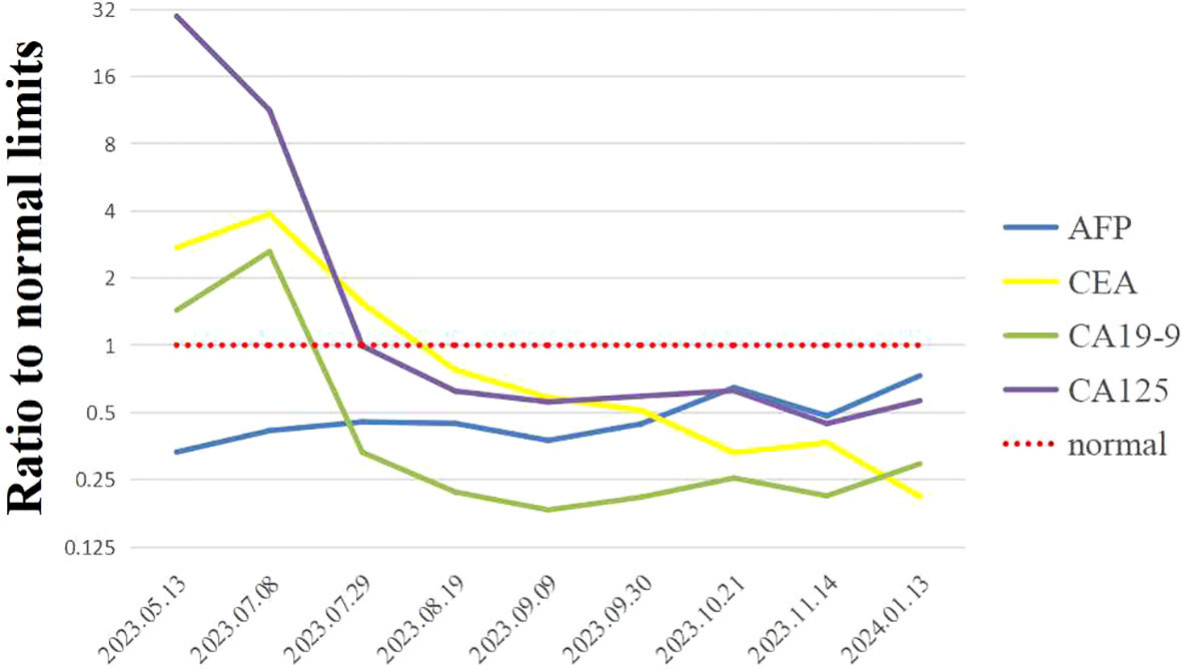
Results of changes in tumor markers of the patient during systemic treatment. Showing ratios to normal limits, including the alpha-fetoprotein (AFP) levels, the carcinoembryonic antigen (CEA) levels, the cancer antigen 19-9 (CA19-9) levels and the cancer antigen 125 (CA125) levels.

A 74-year-old woman was admitted to our hospital with fever and epigastric pain for 10 days. She had a history of cholecystectomy but not chronichepatitis B or C infection or previous diagnosis of neoplasia, with normal limits of total and direct bilirubin levels. Respectively, the cancer antigen 125 level exceeded 1000 ng/ml; the carcinoembryonic antigen level was 13.67 ng/ml; the cancer antigen 19-9 level was 50.2 units/ml; the alpha-fetoprotein level and DCP (Des-gamma-Carboxy Prothrombin) level were within normal limits (**Figure 2**). An upper abdominal enhanced computed tomography (CT) scan showed a slightly lower density of a circular lesion of about 8.3*6.4cm in the right lobe of the liver (**Figure 1B**).

## 3 Diagnostic assessment, details on the therapeutic intervention, follow-up and outcomes

Results of ultrasound-guided liver tissue puncture of the patient were considered to be poorly differentiated adenocarcinoma with necrosis (**Figure 1C**). Immunohistochemical analysis results were as follows: CK7 (+), CK19 (+), CK20 (-), VILLIN (+), Ki-67 (high expression), Vimentin (+). According to the American Joint Committee on Cancer staging system, 8th edition, she was diagnosed with stage IIIB (T2NxM0) iCCA.

**Figure 3:**
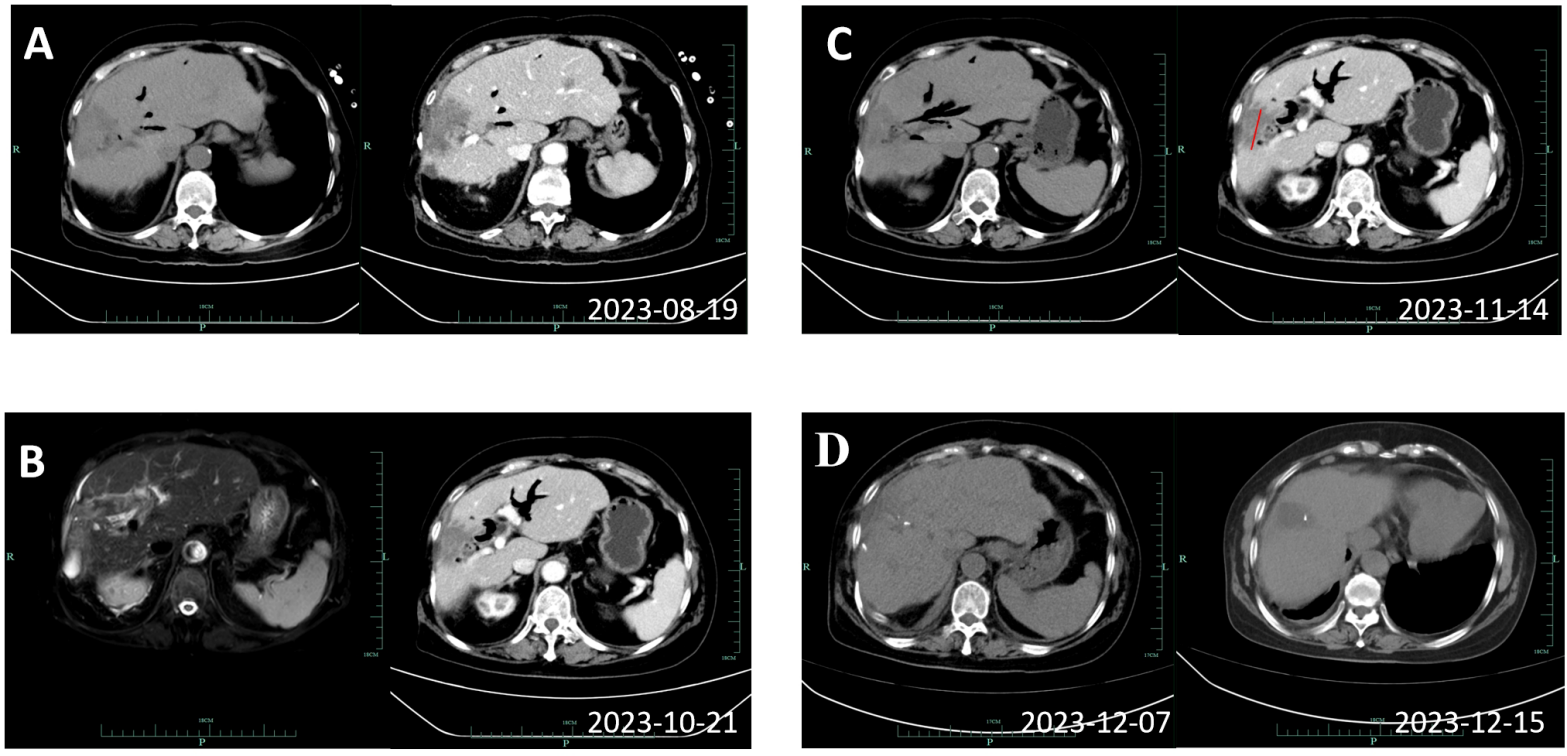
Imaging review results of the patient during systemic therapy. **(A)** Showing enhanced CT results of 2023-08-19, before the third cycle. **(B)** Showing magnetic resonance imaging and cholangiopancreatography results of 2023-10-21, before the sixth cycle. **(C)** Showing enhanced CT of 2023-11-14, after 6 cycles with a reduction in the longest diameter of the tumor to 4.9cm. **(D)** Display the results of the enhanced CT scans of the two postoperative reviews.

According to the opinions discussed by the multidisciplinary team, radical resection is difficult to perform in this patient with a large tumor area. Threfore, the systematic treatment of chemotherapy and immunotherapy was recommended. After 6 cycles of treatment with Gemcitabine (1.6g, d1 and d8), Tegafur (60 mg qm, 40mg qn, d1 to d14), and Zimberelimab (240 mg, q21 days), enhanced CT scans showed a reduction in the maximum tumor diameter from 8.3cm to 4.9cm (**Figures 3A-C**). The treatment response was evaluated to be a partial response according to the revised Response Evaluation Criteria in Solid Tumors (version 1.1). The patient developed myelosuppression and thrombocytopenia during the chemotherapy cycle, which recovered after treatment with elevated white blood cells. No immune-related adverse events were observed.

After re-evaluation of surgical indications, the patient underwent resection of the right hepatic tumor and postoperative T-tube drainage without biliary leakage. No viable tumor cell was detected in the resected specimen; only necrotic tissue was detected, indicating a pathologic complete response after systematic treatment. The postoperative pathology results indicated down staging of the tumor to stage T1aN0M0 without perineural and vessel invasion; the resection margins indicated R0 resection status.

The patient was re-examined by CT on the 5th and 13th day after surgery (**Figure 3D**), which showed the operative area recovered well with no residual lesions. She was discharged on postoperative day 26 with normal daily life and no obvious symptoms of discomfort. In addition, the T tube was removed 11 weeks after surgery. During the regular telephonic and imaging follow-up as of November 2024 every 3 months, the patient informed that she was living a normal daily life without any symptoms or recurrence on imaging. She had therefore achieved disease-free for one year, and we will undergo periodic radiographic follow-up.

## 4 Discussion

In recent years, the incidence and mortality of iCCA have been on the rise worldwide, becoming the second most common primary liver cancer after hepatocellular carcinoma (8), and surgical resection is still the main treatment for iCCA. Due to the relatively insidious clinical symptoms and highly aggressive nature of the disease, about 65% of patients are already in the advanced stage at diagnosis, with median survival of less than 1 year. Surgical resection can increase the median disease-free survival of patients to three years, however, only 20% to 30% of patients can undergo radical surgical resection. In a study of 238 iCCA patients, 54% were considered unresectable at diagnosis, with the most common causes being multifocal liver disease (54%), locally unresectable tumors (29%), and distant disease (13%) (9, 10). Therefore, advanced iCCA can be converted to a surgically resectable level by means of local or systemic therapy, thereby improving patient outcomes. The case we report confirm the conversion of unresectable intrahepatic cholangiocarcinoma in patients with advanced iCCA treated with GS combined with PD-1 inhibitors, demonstrating the safety and efficacy of this regimen.

At present, gemcitabine combined with cisplatin (Gem-Cis) chemotherapy is still the preferred first-line treatment for middle and advanced iCCA. However, C Morizane et al. found that Gem combined S-1 chemotherapy regimens were no less effective than Gem-Cis, with median OS (15.1 months vs13.4 months) and median progression-free survival (PFS) (6.8 months vs5.8 months). At the same time, GC causes nausea, vomiting, anorexia and requires hydration but GS not (11). S-1 (TS-1, Taiho Pharmaceutical) is an oral fluorouracil derivative consisting of tegafur (a prodrug that converts cells to fluorouracil), gemelacil (a dihydropyrimidine dehydrogenase inhibitor that degrades fluorouracil), and oteracil (inhibits phosphorylation of fluorouracil in the gastrointestinal tract), which can be converted into 5-Fu for anti-tumor effect (12). Basides, experimental animal studies on thoracic tumors have shown that S-1 not only inhibits tumor growth, but also initiates a favorable tumor microenvironment, increasing the efficacy of its combined use with immune checkpoint inhibitors (13). In addition, 5-Fu can up-regulate the expression of PD-1 in the tumor microenvironment, being confirmed in gastric cancer, esophageal cancer, colorectal cancer and other tumor disease (14–16).

With the progress of research, the systematic treatment strategy of iCCA has changed from traditional chemotherapy to targeted therapy and immune checkpoint inhibitors. The objective response rate (ORR) benefit of single drug therapy is limited, which makes the combination system therapy schemes emerge in an endless stream. Recently, Li et al. performed 6 cycles of systemic therapy (S-1+ albumin-paclitaxel +PD-1 inhibitor triple regimen) in a patient with IIIB iCCA, followed by successful surgical resection after downphase (17). We conclude the ongoing and completed clinical trials of immuno-chemotherapy for advanced or unresectable iCCA (**Table 1**). As the table shows, in trail phase 1 and 2, patients were treated by the regimens that PD-1/PD-L1 inhibitors combined with GEMOX or Gem-Cis. Meanwhile, primary endpoints were showed by ORR, PFS and conversion rate. Unfortunately, other clinical trials have not provided corresponding main outcomes. In NCT01389414, patients with intrahepatic cholangiocarcinoma treated with panitumumab may have had a survival benefit in comparison with the control group (median PFS was 15.1 vs 11.8 months, P = 0.13) (18).

**Table 1:** Ongoing and completed clinical trials of immuno-chemotherapy for advanced or unresectable iCCA.

Number	Trial phase	Treatment regimens	Number of patients	Primary endpoints
NCT05251662	Phase 2	Sintilimab combined with GEMOX ± IBI305 (Bevacizumab Biosimilar) versus GEMOX	90	Overall response rate
NCT04961788	Phase 2	Toripalimab combined with GEMOX	30	Objective response rate
NCT05781958	Phase 2	Cadonilimab combined with Gem-Cis	60	Objective response rate
NCT04989218	Phase 1 and 2	Durvalumab and Tremelimumab combined with Gem-Cis	1	Objective Response Rate
NCT05738057	Phase 2	Camrelizumab combined with Gem-Cis	22	Conversion rate
NCT04413734	Phase 2	Triprilumab combined with Gem-Cis	120	Progression-free survival
NCT05967182	Phase 2	Pembrolizumab combined with Gem-Cis	24	Progression-free survival; major pathologic response rate
NCT06567600	Phase 2	Pembrolizumab or Durvalumab combined with Gem-Cis	43	Objective response rate
NCT01389414	Phase 2	Panitumumab combined with GEMOX versus GEMOX alone	89	Progression-free survival

GEMOX, gemcitabine and oxaliplatin; HAIC, hepatic arterial infusion chemotherapy; Gem-Cis, gemcitabine and cisplatin.

In summary, we selected a new chemotherapy-combined immunotherapy regimen: gemcitabine +S-1+PD-1 inhibitor. The final results also support our hypothesis that after 6 cycles of conversion therapy, patients with advanced iCCA can successfully decline and undergo surgical resection, with no surviving tumor cells found in postoperative pathologic findings.

As a hot topic in the recent systematic treatment of tumor, our results bring hope for immunotherapy application in the practice of translational therapy for unresectable tumors. However, there are some limitations to this report. First, this report describes only one successful case, and it remains unclear whether other patients are sensitive to this combination regimen, leading to the need for more clinical trials to confirm our findings. Secondly, since the tumor was basically completely necrotic in the postoperative specimens, we could not detect the expression level of PD-1, so there was no practical theoretical basis to confirm whether S-1 might up-regulate the expression of PD-1 in the iCCA tumor microenvironment, and a large number of experimental results were needed to demonstrate this in the future.

## 5 Conclusion

Our results in this case suggest that our regimen that Gemcitabine plus S-1 (GS) combined with PD-1 inhibitors (Zimberelimab) is suitable for converting advanced iCCA to a resectable state; it provides a new treatment option for this tumor. However, as this report describes only one case, future studies with more patients are needed to verify its effectiveness.

## Data Availability

The original contributions presented in the study are included in the article/supplementary material. Further inquiries can be directed to the corresponding author.
